# Deterministic Structural Distortion in Mn^2+^-Doped Layered Hybrid Lead Bromide Perovskite Single Crystals

**DOI:** 10.1021/acsnano.5c08324

**Published:** 2025-07-17

**Authors:** Pushpender Yadav, Kyeongdeuk Moon, Muhammad Shoaib, Puja Thapa, Rui Sun, Seungmin Yang, Jung-Moo Heo, Sijun Seong, John McCracken, Xiwen Gong, Jinsang Kim, Joonho Bang, Dali Sun, Seokhyoung Kim

**Affiliations:** 1 Department of Chemistry, 3078 Michigan State University, East Lansing, Michigan 48824, United States; 2 Department of Physics, 6798 North Carolina State University, Raleigh, North Carolina 27695, United States; 3 Department of Materials Engineering and Convergence Technology, 26720 Gyeongsang National University, Jinju 52828, South Korea; 4 Department of Material Science and Engineering, 1259 University of Michigan, Ann Arbor, Michigan 48109, United States; 5 Department of Chemical Engineering, Department of Electrical Engineering and Computer Science, Macromolecular Science and Engineering Program, Applied Physics Program, 1259 University of Michigan, Ann Arbor, Michigan 48109, United States; 6 School of Materials Science and Engineering, 26720 Gyeongsang National University, Jinju 52828, South Korea

**Keywords:** nanoplatelets, doping, Ruddlesden−Popper perovskite, chemical vapor deposition, distortion, paramagnetic

## Abstract

Dilute magnetic doping in wide-bandgap semiconductors has attracted significant interest due to its potential for tailored optical, spintronic, and spin-photonic properties. While extensive research has explored the optical and magnetic properties of these doped systems, the exact nature of dopant-induced structural properties, particularly in high-quality single crystals, requires further investigation. Here, we demonstrate the synthesis of Mn^2+^-doped (BA)_2_PbBr_4_ (BA=butylammonium) single crystals with well-defined crystal habits and no grain boundaries, enabling controlled investigation into significant crystal deformation as a function of Mn^2+^ incorporation. Structural analysis provides compelling evidence of crystal distortion, manifested by a smooth transition from square nanoplatelets to parallelogram shapes with an in-plane shear distortion of up to ∼6° and an out-of-plane contraction of 9.7% for the highest 4.95% Mn^2+^ concentration. This magnitude of structural change significantly exceeds the typical range observed in doped semiconductors by an order of magnitude. We show, using density functional theory calculations, that the structural distortion upon doping is driven by a thermodynamic energy gain. Static and time-resolved photoluminescence spectroscopy confirms the successful incorporation of Mn^2+^ with characteristic emission at 600 nm with an approximate 0.3 ms radiative lifetime. The uniform incorporation of Mn^2+^ into the host medium is further corroborated by the hyperfine structure in an electron paramagnetic resonance spectrum and the paramagnetic response in superconducting quantum interference device measurements. These findings offer crucial insights into dopant-induced structural modifications, supporting the rational design of dilute magnetic semiconductors for spin-based information technologies.

## Graphical abstract

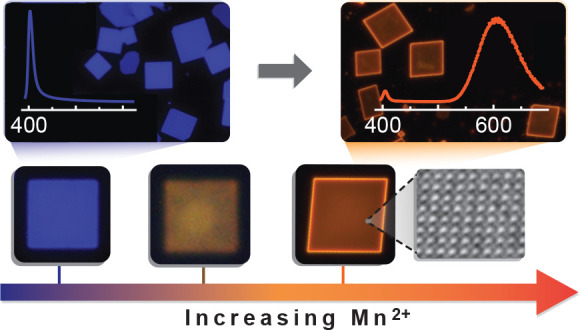

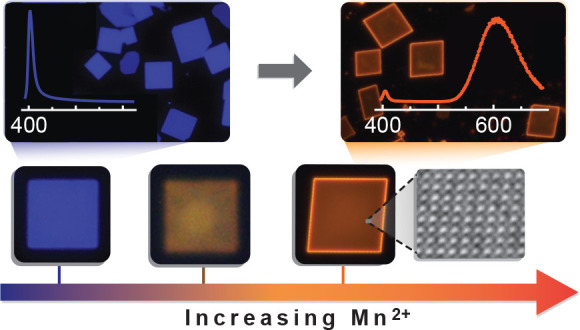

## Introduction

Wide-bandgap semiconductors doped with functional impurities exhibit a range of interesting properties, enabling the development of efficient and controllable building blocks for emerging quantum information technologies in spintronic and spin-photonic applications.^1–5^ The dilute incorporation of Mn^2+^ as a magnetic dopant has extensively been studied with a wide range of highly absorbing semiconductor host materials, including II–VI, III–V, and halide perovskites, to introduce spin degrees of freedom into the existing chip manufacturing infrastructure.^6–9^ Octahedrally coordinated Mn^2+^ in these host media forms a high-spin ground (^6^A_1_) and first excited (^4^T_1_) state,^10,11^ and emission from the spin-forbidden ^4^T_1_–^6^A_1_ transition exhibits a characteristic broad, orange-colored emission band centered around 600 nm with a long radiation lifetime on the order of sub- to few milliseconds.^12–16^ Furthermore, the presence of unpaired electrons in Mn^2+^ has sparked an intense research interest in achieving high-temperature ferromagnetism, making these materials promising candidates for next-generation data storage, quantum computing, and energy-efficient spintronic devices.^17–19^

Recent studies have significantly advanced the understanding in optical, transport and magnetic properties in emerging halide perovskites doped with Mn^2+^.^20,21^ The optical response of Mn^2+^, driven by efficient host-to-dopant energy transfer, shows improved photoluminescence quantum yield (PLQY),^11^ along with tunable radiative transition energy controlled by the strength of crystal fields, making these systems highly promising for solid-state lighting.^15,22^ Identifying the key factors governing the energy *versus* charge transfer to the excited state of Mn^2+^ has proven essential for optimizing dopant emission, directly influencing the efficiency and functionality of optoelectronic applications.^23^ Understanding the incorporation of Mn^2+^ at divalent substitutional *versus* interstitial sites in the perovskite lattice has facilitated the growth of microcrystalline grains with a preferred accumulation of excess Mn^2+^ ions at grain boundaries, enhancing hole extraction and solar cell efficiency in thin films.^24^ Additionally, the presence of unpaired electrons in Mn^2+^-doped perovskites enables parallel spin alignment, resulting in long-range magnetic ordering facilitating ferromagnetism, which has opened up exciting possibilities for spintronic and spin-photonic devices.^18,23,25^

However, despite these prior advancements, challenges remain in achieving sufficient, uniform, and robust incorporation of Mn^2+^ for the development of suitable dilute magnetic semiconductors.^26,27^ While homogeneous and uniform dopant distribution has been thoroughly and accurately characterized in several previous works,^28–34^ it remains ambiguously or insufficiently characterized by a number of other studies, which limits a comprehensive understanding of dopant distribution uniformity and homogeneity. In nanocrystals (NCs) and quantum dots (QDs), uniform incorporation and robust retention of dopants are challenging due to the limited bulk substitution and self-purification of impurities toward the surface. The competition between bulk incorporation and surface doping is a well-known issue,^35–37^ which often results in a significant dopant adsorption on crystal surfaces.^32^ Furthermore, NCs undergo a strong self-purification process, a tendency to expel defects out to the surfaces to minimize their free energy and become defect-free.^35^ This effect is particularly strong in NCs and QDs due to their small sizes because, regardless of their initial position, dopants are always only a few unit cells away from the surface. These surface-segregated dopants may be redissolved away into solvents or often lead to incorrect material characterization such as false magnetic signatures and inaccurate dopant concentrations.^38–41^ Additionally, the inherent size and shape irregularity of QDs pose serious issues, particularly when the property of interest heavily relies on the quantum-confined nature. For instance, the overall linewidth of Mn^2+^ light emission is found to be substantially greater than that of a single QD because of the varying sizes of NCs.^42^ Another, more subtle issue is that not every QD is doped, and a good fraction of QDs may not contain any dopants. Consequently, these factors collectively hinder uniform and robust Mn^2+^ doping in colloidal particles, presenting a fundamental challenge in achieving consistent optical and magnetic properties.

Growing morphology-controlled single crystals presents a promising solution to these challenges as they do not suffer from self-purification or ensemble inhomogeneity. The existing literature on large single crystals of Mn^2+^-doped halide perovskites has demonstrated centimeter- and millimeter-scale crystals,^13,14^ which provide advantages for resolving detailed single-crystal structures and enabling spatiotemporal analyses. However, despite the large sizes, these reports lack direct evidence of true monocrystallinity or well-shaped crystal habits, often with experimental single-crystal X-ray diffraction (SCXRD) results containing missing atoms. In polycrystalline large crystals, grain boundaries and defect sites act as additional locations for dopants in addition to substitutional sites and surfaces.

In both colloidal NCs and large polycrystals, the differing ionic radii (1.19 Å for Pb^2+^ and 0.83 Å for Mn^2+^ under hexa-coordination)^43^ and distinct crystal symmetries preferred by these metal ions (cubic for Pb^2+^ and hexagonal for Mn^2+^) are expected to cause significant structural distortions, typically observed as peak shifts in X-ray diffraction (XRD) patterns. However, many reported XRD patterns reveal only a few tenths of a degree peak shift in both 2D^12–14,12–14,44–48^ and 3D^36,41,49–52^ perovskites, while some examples show no observable dopant-induced crystallographic strain, further raising questions about the dopants’ fate in the host lattices. In layered 2D structures, shifts in the 2θ peak positions, particularly for the (002) and higher-order planes, are indicative of changes in the interlayer spacing (contraction or expansion of the host lattice), which depends on the size difference between the dopant ion and the substituted host cation. The articles reporting small shifts in the XRD peak positions believe that the increase of the 2θ values is consistent with lattice contraction as the decrease of the cation size, due to substitutional doping, shifts the XRD pattern toward higher 2θ values. Considering the reported dopant concentrations on the order of a few % and the ionic size difference, such minimal structural changes are difficult to justify and suggest that dopants are located in other places such as surfaces, grain boundaries, and local domains. As Mn^2+^ dopants in these undesired positions can still yield magnetic and optical responses, it is vital to develop a new synthetic strategy to prepare large single crystals with true monocrystallinity, where Mn^2+^ predominantly occupies substitutional sites. This approach would alleviate uncertainties in dopant positioning and enable a clearer understanding of their impacts on structural, optical, and magnetic properties—ultimately accelerating the development of energy-efficient and high-quality dilute magnetic semiconductor quantum building blocks.

Here, we report a giant and deterministic structural distortion of Mn^2+^-doped layered hybrid perovskite single crystals grown by chemical vapor deposition (CVD). Ruddlesden–Popper phase butylammonium lead bromide ((BA)_2_PbBr_4_ or BAPB, where BA = CH_3_(CH_2_)_3_NH_3_^+^) is used as a host medium, which exhibits defect- and grain boundary-free monocrystallinity with a well-defined square nanoplatelet (NPL) crystal morphology. Upon the introduction of Mn^2+^, BAPB undergoes a significant morphological change from square to a parallelogram shape, with an in-plane angular distortion up to ∼6 ° at the highest Mn^2+^ concentration of 4.95%. Using controlled CVD growth,^53^ we demonstrate a systematic change in the dopant concentration and resulting crystallographic deformation as a function of the Mn^2+^ precursor loading ratio. For the out-of-plane stacking structure, our XRD analysis shows the maximum shift of 0.69° for the fundamental (002) plane at 6.41°, equivalent to a near 10% interlayer contraction, which is over an order of magnitude greater than any deformation observed in the literature of similar systems. This lattice distortion was also observed similarly in hexylammonium lead bromide (HA_2_PbBr_4_ or HAPB), which contains a longer cation chain, indicating that this dopant-induced lattice distortion is a general phenomenon. Theoretical density functional theory (DFT) calculations suggest that this dramatic crystallographic distortion is driven by a thermodynamic energy gain resulting from a reduction in both the unit cell volume and the average bond distance (*d*_avg_). Static and time-resolved photoluminescence (PL) spectroscopy reveals a broad emission envelope centered around 600 nm with an approximately 0.3 ms radiative lifetime, which is characteristic of hexa-coordinated Mn^2+^. The uniform incorporation of Mn^2+^ into the host medium is further corroborated by the hyperfine structure of an electron paramagnetic resonance (EPR) spectrum and a paramagnetic response from superconducting quantum interference device (SQUID) measurements. Our finding presents a promising avenue for potentially achieving an even higher bulk incorporation of Mn^2+^ in large-area thin films with long-range magnetic ordering in ambient conditions.

## Results and Discussion

### Synthesis and Structural Analysis

Figure 1 presents a progressive change in the crystal morphology with incresing dopant precursor loading. A Mn-to-Pb loading ratio, σ, is defined as the molar ratio of Mn^2+^ to Pb^2+^ loaded into the CVD reactor (see Methods for detailed experimental conditions). A pure BAPB NPL with an orthorhombic unit cell^54–56^ typically exhibits a square shape with an in-plane axial angle γ = 90° when imaged along normal to the substrate, as illustrated in Figure 1A. The optical dark-field (DF) image in Figure 1E shows a strong scattering along the edges of the NPL with little intensity from top surfaces, indicating that the NPLs have nearly atomically smooth surfaces (Figure S4 for the AFM topology map). The violet-colored PL is observed uniformly throughout the crystal. Upon slightly increasing σ to 0.03, we observed the PL color changing from violet to pale orange (Figure 1B,F). In this very dilute condition, while no significant morphological change is yet observed, the change in the PL color indicates the presence of a small amount of substitutional Mn^2+^. The hazy surface and nonuniformity observed in DF and PL, respectively, suggest that the growth was slightly perturbed with the introduction of Mn^2+^ precursors and caused an inhomogeneity in crystal surfaces.

**Figure 1:**
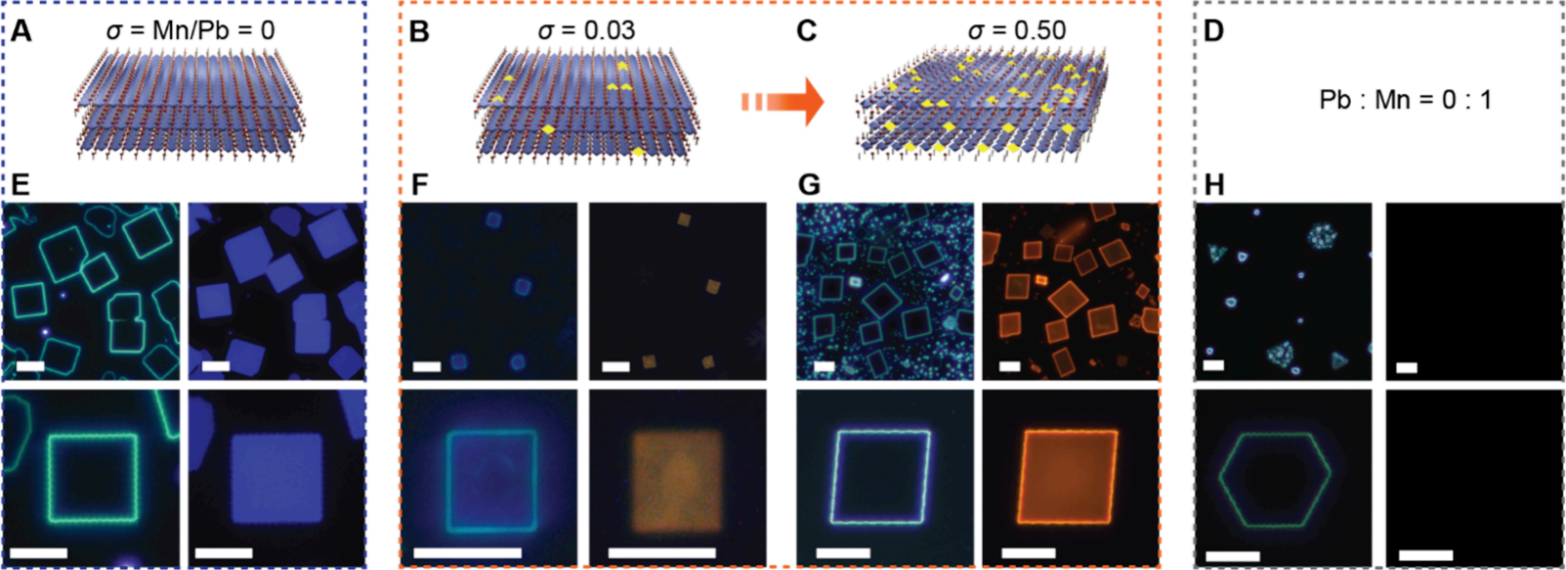
Mn^2+^-doped BAPB NPLs. (A–D) Schematic illustration of progressive change from pure BAPB with zero Mn-to-Pb precursor loading ratio (σ) (A) to doped BAPB with σ = 0.03 (B) and 0.50 (C) and finally to all Pb^2+^ replaced with Mn^2+^ (D). (E–H) Optical dark-field (DF, left) and PL (right) images of NPLs corresponding to the models shown above with σ = 0 (E), 0.03 (F), and 0.50 (G) and fully substituted NPL (H); all scale bars, 10 μm.

A significant change was observed when σ was further increased to 0.50. All NPLs, with clean surfaces and well-defined crystal habits, exhibited γ clearly different from 90°. Additionally, substantially more intense vivid orange PL was observed (Figure 1C,G). The stronger PL originates from the increased concentration of the Mn^2+^ emission centers. However, such a large lattice deformation was rather surprising and unprecedented in Mn^2+^-doped semiconductor systems. To make a clear comparison with an extreme case, we prepared crystals with all of the Pb^2+^ replaced with Mn^2+^ (Figure 1D). At complete Mn^2+^ substitution, NPLs with hexagonal morphologies were observed (Figure 1H), which we presume to be (BA)_2_MnBr_4_. Unfortunately, (BA)_2_MnBr_4_ is extremely hygroscopic similar to the three-dimensional (3D) analog CsMnBr_3_ ^57–59^ and would not survive in an ambient condition for further characterization. The low-magnification image in Figure 1H shows clusters of small semispherical particles surrounded by either a hexagonal or triangular outer trace, suggesting that (BA)_2_MnBr_4_ NPLs were initially formed and quickly degraded by capturing moisture from the air. From this series of syntheses, we draw a qualitative hypothesis that when Mn^2+^, which prefers to form a hexagonal crystal symmetry when present in a sufficiently large amount, is incorporated into a square-forming BAPB layer, it will impose a substantial lattice stress and consequently result in a nontrivial crystal deformation.

For a comprehensive examination of dopant-induced crystal distortion, we employed electron microscopy and XRD analysis to probe separately the in-plane and out-of-plane crystal structures, respectively. First of all, we analyzed the actual incorporation concentration, *x* in the chemical formula (BA)_2_Mn_*x*_Pb_1–*x*_Br_4_, as a function of σ. Shown in Figure 2A are a representative scanning transmission electron microscope (STEM) image and energy-dispersive X-ray spectroscopy (EDS) elemental maps acquired from a sample grown with σ = 0.50. These STEM images show a clear off-90° axial angle and homogeneous distribution of Pb, Br, and Mn. The *x* value, an actual concentration of Mn^2+^ relative to Pb^2+^, from this crystal was measured at 0.045. A series of *x* was obtained from 10 individual NPLs from four different batches with (i) σ = 0, (ii) 0.03, (iii) 0.15, and (iv) 0.50. The result revealed that (i) *x* = 0, (ii) 0.010 ± 0.002, (iii) 0.018 ± 0.003, and (iv) 0.045 ± 0.006, which is plotted in Figure 2B, with a good positive correlation between *x* and σ. Corners of single-crystal NPLs from each of the four batches were imaged using scanning electron microscopy (SEM) and are presented in Figure 2C to highlight the progressive change in the axial angle γ. While the change in γ (Δγ = γ – 90°) is not easily noticeable for the lowest *x* = 0.01, it becomes more apparent as *x* increases. Quantitative image analysis over 30 different NPLs allowed the precise determination of Δγ, as displayed in Figure 2D. With the highest *x* = 0.049, we observed an unprecedented in-plane distortion as large as 5.72°. To verify that the morphological Δγ indeed arises from deformed atomic arrangements as opposed to higher-index planes, we acquired an atom-resolved image of a (BA)_2_Mn_0.045_Pb_0.955_Br_4_ NPL using aberration-corrected STEM high-angle annular dark-field (HAADF) imaging. Despite the high beam sensitivity of these layered hybrid perovskite NPLs, we were able to obtain a good atomic image from repeated rounds of experiments. The projected image along the (001) zone axis reveals atomic arrangements consistent with the Ruddlesden–Popper phase structure^60^ with a clear nonzero Δγ (Figure 2E). Fast Fourier transform (FFT) analysis (inset of Figure 2E) corroborates the periodic atomic arrangements along different crystallographic directions.

**Figure 2:**
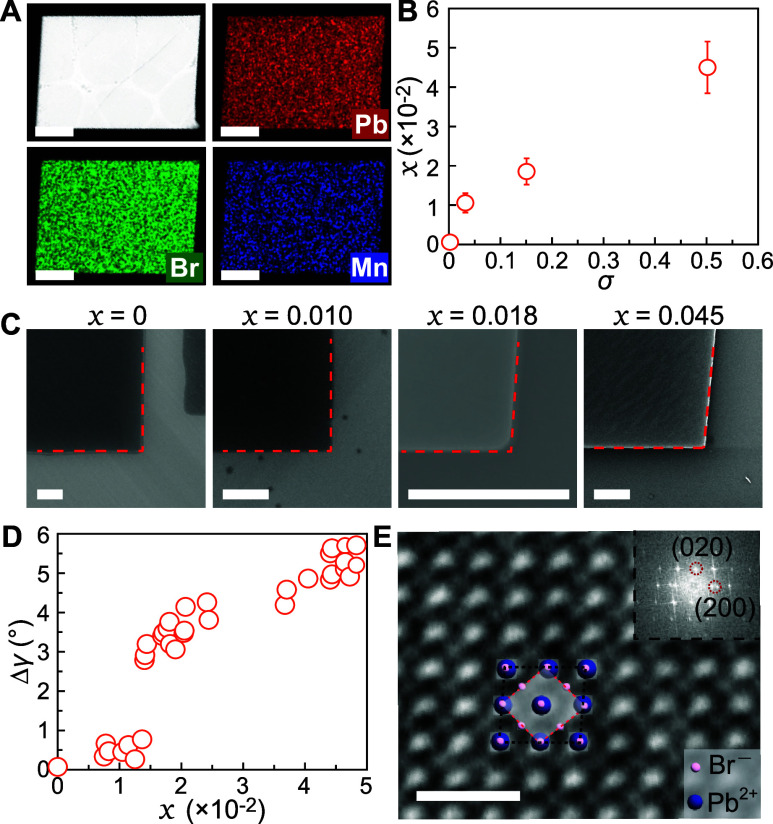
Electron microscopy analysis of the in-plane crystal structure. (A) STEM image (white) of (BA)_2_Mn_*x*_Pb_1–*x*_Br_4_ with *x* = 0.045 and elemental maps for Pb (red), Br (green), and Mn (blue); scale bars, 1 μm. (B) Dopant incorporation concentration (*x*) as a function of σ. (C) SEM images of NPL corners highlighting the angle change with increasing *x*. (D) Statistical analysis of the angle distortion (Δγ = γ – 90°) as a function of *x* measured from 30 individual NPLs. (E) STEM-HAADF atom-resolved image of *x* = 0.045 NPL; scale bar: 1 nm. Inset: fast Fourier transform pattern with select planes assigned.

The out-of-plane layering structure is examined using powder XRD. The XRD patterns of NPL ensembles with three different concentrations are presented in Figure 3A. All three patterns primarily exhibit (002) reflections and their higher orders, indicating phase-pure, highly oriented CVD growth with perovskite layers lying parallel to the growth substrate.^53^ The first primary peaks around 6.5° were magnified in Figure 3B, where we observed an unusually large peak shift. For *x* = 0, the fundamental (002) reflection appears at 2θ = 6.41°, consistent with previous reports.^12,53^ At *x* = 0.01, the peak shifts to 6.53°, corresponding to a relative peak shift (δ = Δ2θ/2θ) of 1.5%. This δ already exceeds the highest degree of distortion commonly reported for Mn^2+^-doped halide perovskites.^12–14,36,41,12–14,44–47,49,50^ At the highest *x* = 0.045, the fundamental peak shifts to 7.1° with a Δ2θ = 0.69°, which corresponds to an even greater δ of 10.9%. The magnitude of crystal distortion observed under this condition far exceeds the prior observations by an order of magnitude. It suggests that a profound structural reorganization occurs with a high substitutional Mn^2+^ incorporation. Analysis of the (002) interplanar spacing reveals a systematic reduction from 13.77 Å (for *x* = 0) to 13.51 Å (*x* = 0.010) and 12.43 Å (*x* = 0.045) (Figure 3D), showing a giant interlayer contraction by 9.7%. This substantial compression of the interlayer spacing is attributed to the smaller ionic radius of Mn^2+^ (0.83 Å) compared to Pb^2+^ (1.19 Å).^43^

**Figure 3:**
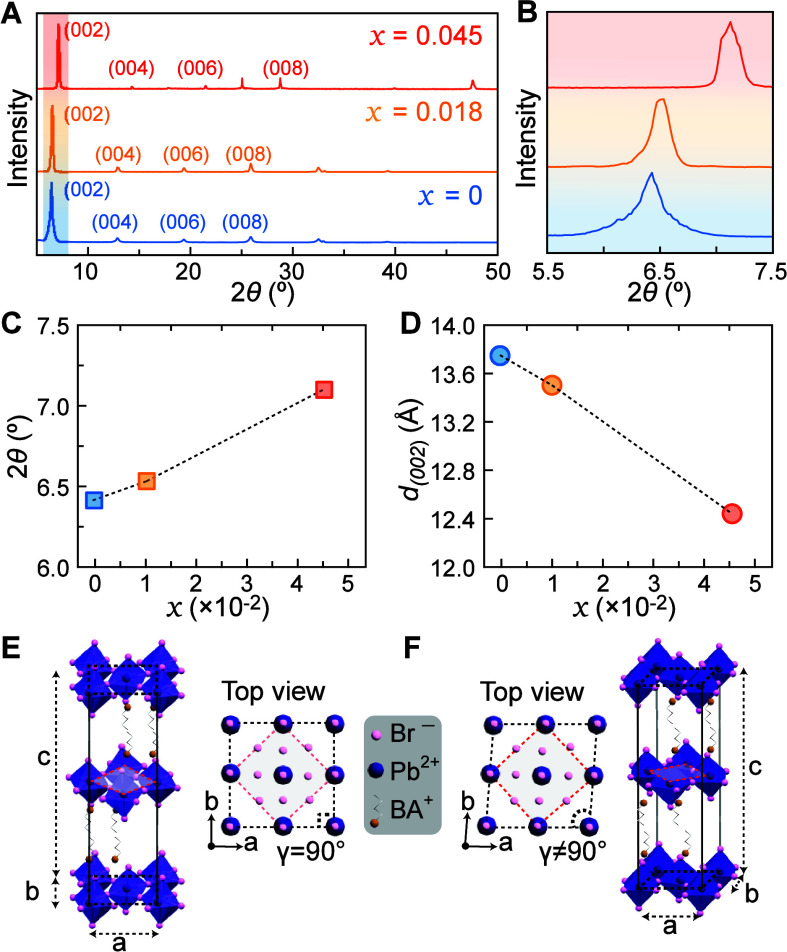
XRD analysis of the lattice stacking structure. (A) XRD patterns with *x* = 0 (bottom), 0.018 (middle), and 0.045 (top). (B) The magnified view around the (002) peak. (C, D) 2θ peak position (C) and calculated interlayer spacing (D) of (002) plane as a function of *x*. (E, F) Schematic representation of the orthorhombic unit cell of pure BAPB (E) and distorted unit cell of (BA)_2_Mn_*x*_Pb_1–*x*_Br_4_ (F), each with a projected view along the *c* axis.

Combining in-plane and out-of-plane structural analyses, we propose an orthorhombic-to-monoclinic unit cell transition upon Mn^2+^ incorporation. While the cell parameters for pure orthorhombic BAPB are well-defined with *a* = 8.3 Å, *b* = 8.2 Å, *c* = 27.5 Å, and α = β = γ = 90°,^13,14^ the unit cell parameters for monoclinic (BA)_2_Mn_*x*_Pb_1–*x*_Br_4_ vary with *x*. These parameters must be defined for a given *x* of interest, as discussed throughout the structural analysis. For instance, *a* = 8.1 Å, *b* = 8.0 Å (Figure S9), *c* = 24.8 Å, α = β = 90°, and γ = 94.8° for *x* = 0.045, obtained from the atomic STEM-HAADF image and XRD pattern. This level of strain effect is unprecedented in prior studies of doped nanocrystals, QDs, and large crystals, where structural changes were either less pronounced or negligible. As previously reported, even upon using a very high Mn^2+^ precursor concentration (several hundred % Mn^2+^ loading relative to Pb^2+^) in the reaction mixture solution, only 0.6% of Mn^2+^ was found to be incorporated into the crystal.^45^ This difficulty of achieving significant Mn^2+^ incorporation has been widely observed in the literature. As a result, it is believed that Mn^2+^ more readily occupies edge sites, surface sites, or grain boundaries rather than interior lattice sites. While the presence of Mn^2+^ on the surfaces can still yield desired properties, such as long-lived PL, they often fail to influence the global structure, instead allowing the host lattice to maintain its original configuration, resulting in no XRD peak shift.

Our study suggests that irreversible vapor-phase trapping of dopants may have led to improved homogeneity of substitutional doping relative to previous approaches. Although the *apparent* Mn^2+^ concentration in our work is not significantly greater than the values reported in the literature,^13,14^ a high level of substitutional Mn^2+^ incorporation was achieved due to the highly kinetic nature of the CVD process.^61^ Unlike solution methods, CVD often produces materials with nonequilibrium characteristics in dopant concentration,^62^ crystal structure,^63^ and morphology.^53^ Specifically, we believe that predominant substitutional doping into the bulk of the crystals was enabled by the irreversible kinetic trapping of dopants during growth.^62,64,65^

Additionally, the mechanical softness of the layered hybrid BAPB host may have played a role in achieving a high substitutional Mn^2+^ concentration. The elastic constant of 2D BAPB is 8.82 GPa,^66^ substantially lower than that of all-inorganic 3D CsPbBr_3_ (16 GPa)^67,68^ or conventional III–V compounds (123 GPa for GaAs).^69,70^ We performed a comparison experiment in which the same Mn^2+^ doping was applied to a stiffer 3D CsPbBr_3_. The quality and morphology of Mn^2+^-doped CsPbBr_3_ crystals were severely destroyed (see Methods for detailed experimental conditions), yielding irregularly shaped round particles that were characterized as PbBr_2_ crystals by the XRD pattern (Figure S10). Thus, the large lattice strain caused by Mn^2+^ is better accommodated within the softer BAPB lattice compared to 3D CsPbBr_3_. Here, it is also important to note that an inhomogeneous distribution of Mn^2+^ emission colors was observed from the irregular Mn^2+^-doped PbBr_2_, ranging from orange to yellow. It demonstrates that exciton energy transfer to Mn^2+^ does not necessarily require a high crystal quality. Therefore, the observation of Mn^2+^ emission alone cannot be direct evidence of ideal substitutional incorporation.

To verify whether the observed crystal distortion is a general phenomenon across the 2D perovskite systems, we conducted comparable doping experiments using hexylammonium as the long-chain cation, forming hexylammonium lead bromide ((HA)_2_PbBr_4_ or HAPB). Figure 4A displays the DF and PL images with increasing σ from 0 (leftmost) to 0.5 (rightmost). Undoped HAPB exhibits a remarkably smooth single NPL in the DF image and a blue PL, consistent with previous reports.^71–73^ Upon the introduction of Mn^2+^ into the reaction chamber at a low concentration (σ = 0.10), we started observing the characteristic Mn^2+^ emission. As we increased σ from 0.10 to 0.50, a similar trend of progressively increasing in-plane distortion emerged, accompanied by more intense orange-yellow emission (see Figure S11 for more low-magnification images). Figure 4B presents a quantitative in-plane distortion, Δγ, as a function of σ, which exhibits a strong positive correlation. To characterize the out-of-plane structure, we performed XRD analysis at different σ values as shown in Figure S12. Figure 4C displays the magnified primary peaks originally observed around 5.0°, revealing a systematic shift toward higher 2θ angles with increasing Mn^2+^ concentration, a trend that we observed with BAPB. For undoped samples (σ = 0), the fundamental (002) reflection appears at 2θ = 4.92°. At σ = 0.10, this peak shifts to 4.98°, corresponding to an δ of 1.2%. As we increased the dopant concentration to σ = 0.30, the peak further shifted to 5.12°, yielding a more substantial shift of δ = 4.0%. At our highest doping concentration (σ = 0.50), the synthesis of doped HAPB was significantly disrupted and did not produce a sufficient yield and purity of desired crystals (Figure S12), preventing us from obtaining an ensemble XRD pattern. Nevertheless, the clear progressive shifts observed up to σ = 0.30 provide compelling evidence of systematic crystallographic changes induced by Mn^2+^ incorporation that closely parallel our observations in the Mn^2+^-doped BAPB system. We performed additional doped NPL growth on amorphous SiO_2_ and crystalline C-plane sapphire substrates to demonstrate that structural distortion is not a substrate-directed phenomenon. As shown in Figure S13, crystal distortions were observed on both substrates, confirming no correlation between doping-induced distortion and the nature of the underlying substrates.

**Figure 4:**
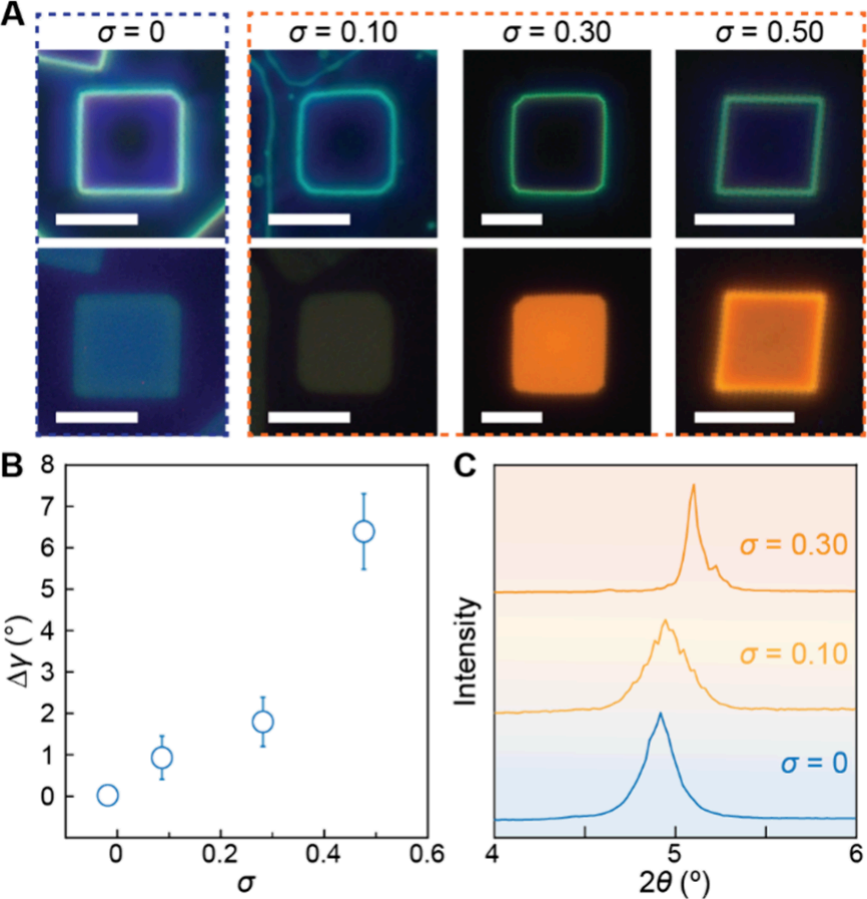
Mn^2+^-doped HAPB NPLs. (A) Optical DF (top row) and PL (bottom row) images of HAPB NPLs corresponding to σ = 0, 0.10, 0.30, and 0.50 (left to right); all scale bars, 5 μm. (B) Statistical analysis of Δγ as a function of σ measured for 10 NPLs of each σ. (C) The magnified view around the (002) XRD peak with σ = 0 (bottom), 0.10 (middle), and 0.30 (top).

Theoretical first-principles calculations were performed to identify the thermodynamic origin of the lattice strain by comparing distorted structures with undistorted, yet doped, structures. An orthorhombic BAPB unit cell was considered with the center Pb^2+^ ion substituted by a Mn^2+^ ion (Figure 5A), and structural relaxation was conducted under two conditions: (i) with the orthorhombic crystal symmetry fixed (maintaining α = β = γ = 90°) and (ii) with no structural constraints. We observed that the unit cell, when relaxed without constraints, distorted away from the orthorhombic structure (Figure 5B) with the cell volume reduced by 1.5% compared to the constrained orthorhombic unit cell (Table S1). Notably, the distorted structure showed deviations in the lattice angles from 90° (left panel of Figure 5B), reproducing the shear distortion observed experimentally. Moreover, the relaxed distorted structure resulted in an ∼50 meV decrease in the total energy per unit cell compared to the nondistorted orthorhombic structure, suggesting that the lattice distortion is driven by thermodynamic energy stabilization (right panel of Figure 5B).

**Figure 5:**
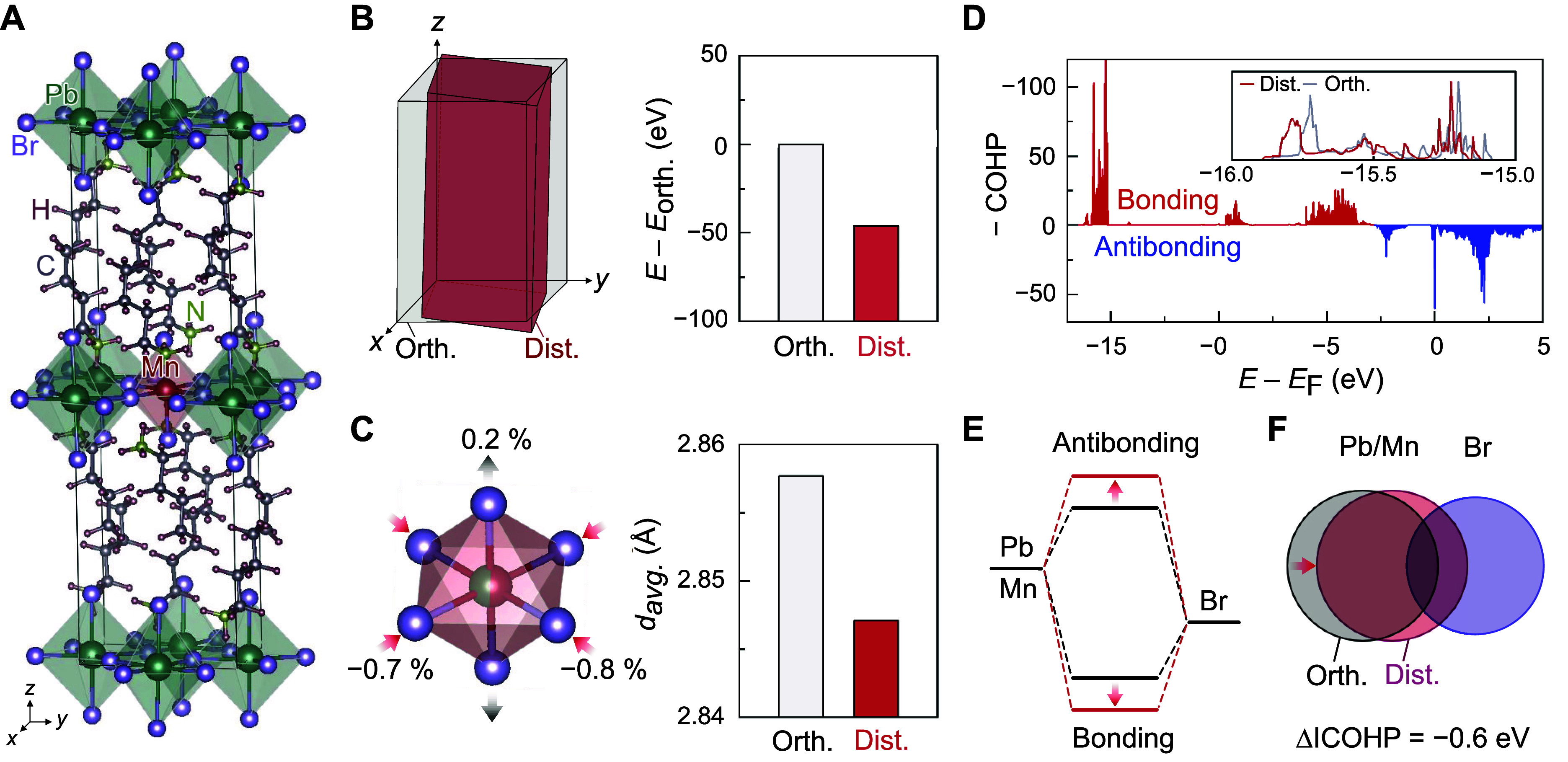
Effects of Mn^2+^ substitution on lattice distortion. (A) Unit cell of BAPB with the center Pb^2+^ substituted by Mn^2+^. (B) Unit cell distortion obtained from the relaxation of the Mn^2+^-doped orthorhombic unit cell (left) and the total energy difference between orthorhombic and distorted structures (right). (C) Changes in the local bonding environment and *d*_avg_ of the (Pb/Mn)­Br_6_ octahedron in orthorhombic and distorted structures. (D) Crystal orbital Hamilton population (COHP) of the Pb/Mn–Br bonds. The red and blue colors represent bonding and antibonding characters, respectively. The inset compares COHP curves for the orthorhombic and distorted structures. (E, F) Schematic illustrations of the increased energy splitting between the bonding and antibonding molecular orbitals (E) and enhanced orbital overlap between metal and halide ions, with a calculated change in integrated COHP (ΔICOHP) (F).

To further investigate the origin of thermodynamic stabilization, we examined the changes in the bond characteristics between the metal cations and halide anions. The lattice distortion caused a reduction in bond distances between Pb/Mn cations and surrounding Br anions by −0.7, −0.8, and 0.4% along the *x*, *y*, and *z* axes, respectively, resulting in an overall decrease of the *d*_avg_ (Figure 5C). This decrease in *d*_avg_ induces two important changes in bond characteristics. First, the bond length contraction increases the energy splitting between the bonding and antibonding molecular orbitals. Figure 5D shows the crystal orbital Hamilton population (COHP) curves of the Pb/Mn–Br bonds in the distorted structure, where fully occupied bonding states appear below −2.7 eV and partially occupied antibonding states appear above −2.7 eV. The structural distortion shifts the bonding and antibonding molecular orbitals to lower and higher energies, respectively (inset of Figure 5D,E and Figure S14). Second, the bond strength is enhanced as the bond length decreases due to an increased orbital overlap between cations and anions. The integrated COHP value decreased by 1.4% during structural distortion, indicating that the bonds between the cations and anions were strengthened (Figure 5F).

From the theoretical calculation, we summarize that the partial substitution of larger Pb^2+^ with smaller Mn^2+^ in the BAPB lattice results in a reduction in both the unit cell volume and *d*_avg_ between the metal and halide ions. This reduction in *d*_avg_ strengthens the bonds overall (Figure 5F), ultimately leading to thermodynamic stabilization. We note that the calculated values in this theoretical approach are not quantitatively accurate, as the periodic expansion of the monosubstituted unit cell corresponds to the doping level of 25%, far greater than the highest Mn^2+^ concentration (4.95%) experimentally achieved in this study. A more accurate representation of experimental dopant concentrations requires a much larger supercell, but this construction is limited by the large number of atoms (156 atoms per unit cell) present in a single BAPB unit cell. Nevertheless, this theoretical framework provides foundational insight into the origin of the giant structural distortion.

### 2 Optical Properties

The luminescent properties and energy transfer behaviors of BAPB and (BA)_2_Mn_0.045_Pb_0.955_Br_4_ NPLs were investigated. Figure 5A,B displays the PL and photoluminescence excitation (PLE) spectra of undoped and doped NPLs. Undoped NPL shows a narrow PL peak centered at 410 nm, and the PLE spectrum reveals a peak at ∼405 nm, corresponding to the bandgap of BAPB. It indicates that the blue 410 nm emission is due to the formation of free excitons (FEs) with a small Stokes shift. Upon 4.5% Mn^2+^ doping, the FE emission is significantly suppressed and remains unchanged, while a broad emission band centered at 600 nm strongly emerges (Figure 6B). Although DFT calculations suggest an increasein the bandgap upon Mn^2+^ insertion, the predicted change of 0.6 eV corresponds to a 25%-doped model system. In contrast, our experimentally calculated doping level is only 4.5%, which would result in an even smaller bandgap change. Therefore, the FE emission peak is expected to be indistinguishable from that of the undoped system. The broad 600 nm emission is characteristic to the ^4^T_1_–^6^A_1_ *d–d* transition of octahedrally coordinated Mn^2+^ ions in the BAPB host. The PLE spectrum collected at 600 nm (Figure 6B) closely mirrors that of undoped BAPB, indicating that the 600 nm emission is sensitized by interband absorption of the host. This clearly shows an efficient energy transfer mechanism from the host perovskite to the Mn^2+^ dopants. The features observed beyond the excitonic peak in the PLE spectra differ between the undoped and doped systems because the measurements were conducted at two different emission wavelengths of 430 and 614 nm, respectively. These wavelengths correspond to distinct emission pathways: radiative recombination of FEs of the host lattice at 430 nm and the *d–d* transition of Mn^2+^ at 614 nm. This suggests that the energy transfer from the host lattice to the Mn^2+^ dopant is more efficient from the first excited state than from higher-order excited states, which explains the lower intensity of the second excitation band in the PLE spectra. As a result, the excitation profiles reflect the differing energy transfer pathways and efficiencies in the undoped and doped system. Overall, no significant change in the optoelectronic properties of the BAPB host was observed experimentally with dopant-induced distortions, which is consistent with predictions from the band structure calculations (COHP in Figure 5D). This aligns with a prior study reporting that, in layered hybrid perovskites, in-plane bond distortions up to ∼30° do not result in as significant changes in luminescent properties as those caused by out-of-plane lattice buckling.^54^

**Figure 6:**
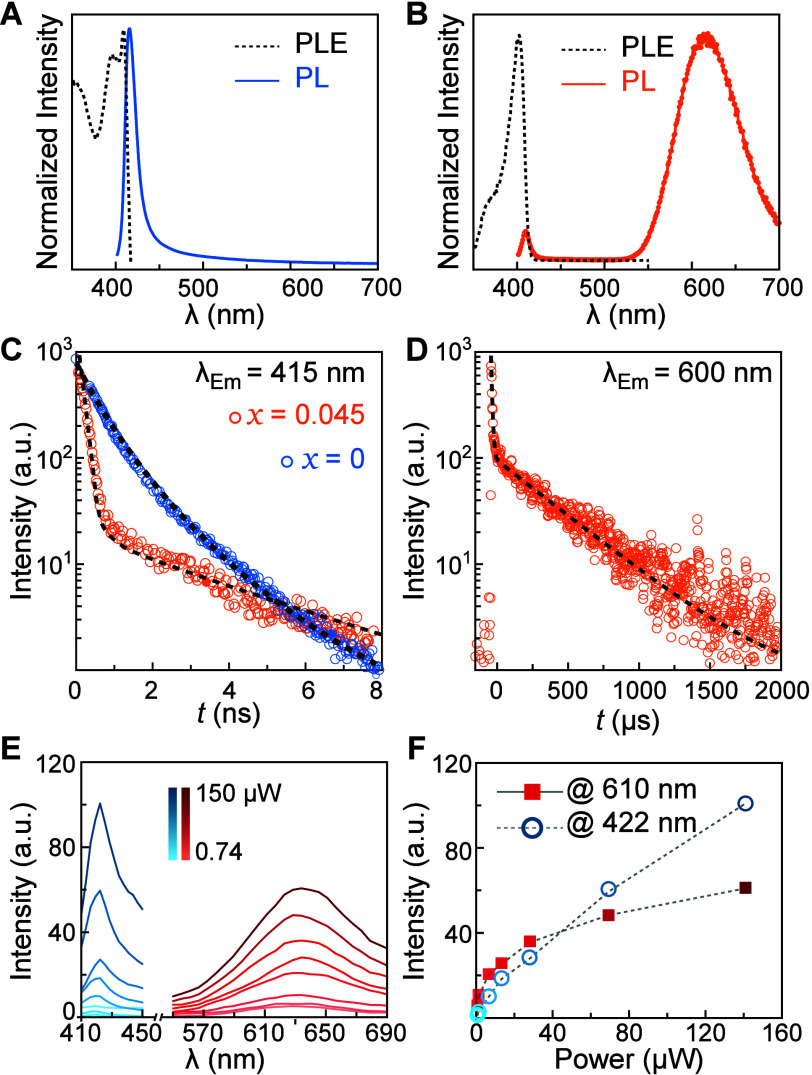
Optical properties. (A, B) PL and PLE spectra of (BA)_2_Mn_*x*_Pb_1–*x*_Br_4_ with *x* = 0 (A) and *x* = 0.045 (B). (C) Time-resolved PL spectra measured at 415 nm for *x* = 0 (blue) and *x* = 0.045 (orange) fitted to biexponential functions (black dashes). (D) Time-resolved PL spectra of (BA)_2_Mn_0.045_Pb_0.955_Br_4_ excited and measured at 400 and 600 nm, respectively, with a fit to a biexponential function (black dashes). (E) Power-dependent PL spectra of (BA)_2_Mn_0.045_Pb_0.955_Br_4_. (F) Change in the intensity of FE emission at 422 nm (blue circles) and Mn^2+^ emission at 610 nm (red squares) as a function of excitation power.

The radiative lifetimes of the FE and Mn^2+^ emissions were examined using time-resolved PL spectroscopy. For pure BAPB, the FE emission showed a radiative lifetime of ∼1.0 ns, which is in close agreement with the reported data (Figure 6C).^53^ For (BA)_2_Mn_0.045_Pb_0.955_Br_4_, two distinct decay times were observed with 0.3 and 3.2 ns, of which the accelerated decay is likely caused by dopant-to-host energy transfer and distortion-induced nonradiative recombination pathways, which are commonly observed in materials with defects and grain boundries.^45^ In contrast, the slower decay component corresponds to the intrinsic radiative recombination of FEs within the host lattice. The emission band at ∼600 nm shows a characteristic long 378 μs decay time following a short 7.2 μs decay component (Figure 6D). The short decay process indicates the presence of possible Auger recombination or unwanted energy back-transfer to the host lattice. The decay fitting parameters can be found in Table S2.

To elucidate the nature of the energy transfer process and saturation behavior, we conducted power-dependent PL measurements using a confocal laser microscope (Figure 6E). Excitation power was varied from 0.74 to 150 μW by using a 405 nm laser focused to a diffraction-limited spot size and rapidly scanned across the sample with a 3.2 μs dwell time per position. The observed increase in the overall PL envelope indicates the absence of additional photophysical processes within the measured power range. Analysis of the integrated PL intensity as a function of excitation power density (Figure 6F) reveals distinct regimes of behavior. The FE intensity shows a good linearity with increasing power, indicating there is no extrinsic saturation effect. For the Mn^2+^ emission, however, we observe a linear dependence at low power densities below ∼20 μW, which enters a sublinear regime as power density increases. This is attributed to the finite number of external Mn^2+^ dopants available for capturing electrons from the host.

### 3 Magnetic Properties

We examined the magnetic properties of Mn^2+^-doped BAPB NPLs. EPR spectroscopy has been carried out at *T* = 130 K to identify the presence of isolated, noninteracting Mn^2+^ ions in doped BAPB NPLs. The EPR spectrum displayed in Figure 7A exhibits a clear hyperfine splitting with a splitting constant of ∼89 G. Comparing with the reported hyperfine splitting constants from the literature for Mn^2+^-doped hybrid perovskites, we confirmed that Mn is present in the 2+ oxidation state at the Pb^2+^ site in a dilute amount.^9,50^

**Figure 7:**
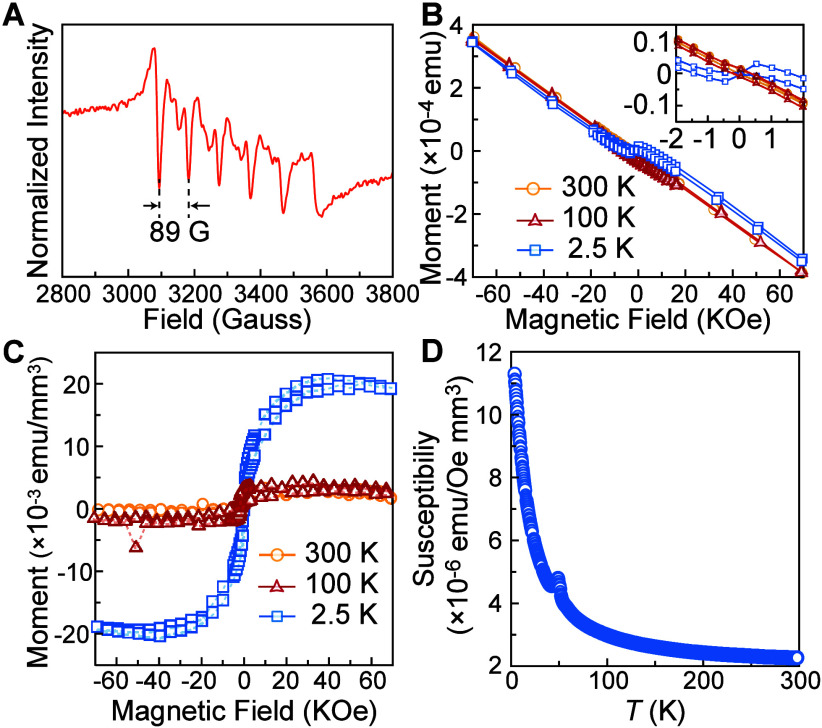
Magnetic properties of (BA)_2_Mn_0.045_Pb_0.955_Br_4_ NPLs. (A) EPR spectrum measured at *T* = 130 K. (B) Field-dependent magnetization *M*–*H* curves measured at different temperatures, including diamagnetic responses from substrates. The inset shows the *M*–*H* curves within the range of ± 2 kOe. (C) *M*–*H* curves after subtracting the substrate contribution at different temperatures. (D) Temperature dependence of the magnetic moment of (BA)_2_Mn_0.045_Pb_0.955_Br_4_ in an applied magnetic field of 7 T under ZFC condition.

To characterize the magnetic activity of Mn^2+^ dopants, we performed SQUID measurements on (BA)_2_Mn_0.045_Pb_0.955_Br_4_ NPLs (see Methods for details). Figure 7B shows the *M*–*H* loops obtained at *T* = 2.5, 100, and 300 K. It is noteworthy that there is a negative linear background under the applied magnetic field, together with a tilted “S” shape at *T* = 2.5 K as shown in the inset. This negative linear background originates from the diamagnetic response of the silica substrate (∼0.4 mm thick). After the background subtraction, the “S” shape of the *M*–*H* loop appears clearer at 2.5 K as shown in Figure 7C, consistent with the expected paramagnetic nature in doped (BA)_2_Mn_0.045_Pb_0.955_Br_4_. The paramagnetic behavior of (BA)_2_Mn_0.045_Pb_0.955_Br_4_ is further supported by the *M*–*T* curve shown in Figure 7D. We measured the magnetic moment over the temperature range of 2.5 to 300 K at an applied external field of 7 T under zero-field cooling (ZFC) condition. The result shows a monotonous increase of magnetic moment with decreasing temperature, which can be attributed to the suppression of thermal excitation that destroys the spin alignment.^74,75^ The presence of hyperfine splitting, the “S” shape of the *M*–*H* loop, and the *M*–*T* curve all confirm the paramagnetic nature in the doped (BA)_2_Mn_0.045_Pb_0.955_Br_4_.

## Conclusions

In conclusion, we demonstrated a significant effect of Mn^2+^ substitution on the crystal distortion of BAPB and HAPB using grain boundary-free NPL single crystals. Using controlled CVD growth, we achieved a remarkable degree of lattice distortion of Mn^2+^-doped BAPB and HAPB, far exceeding the structural changes observed in doped semiconductor systems. A direct correlation between Mn^2+^ substitution and the magnitude of lattice deformation was revealed by systematic analysis of in-plane (Δγ) and out-of-plane (Δ2θ) structural parameters, as well as energetics calculations. The dilute presence of Mn^2+^ was supported by spectroscopic and magnetic measurements. The synthetic approach and comprehensive analysis demonstrated in this work establish a blueprint for exploring dopant-induced transformations across a broader range of semiconductor systems, paving the way to addressing emerging spintronic and spin-photonic devices.

## Methods

### Growth Substrates and Cleaning Process

NPLs were grown on a SiO_2_ wafer (Nova Electronic Materials; n-type (100) Si with 3000 Å thermal oxide; 1–10 Ω-cm) or microscope glass slide or C-pane sapphire cut in 1 × 2 cm size in our home-built CVD system in which all control parameters are automatically regulated by our custom-written program. Growth substrates were first cleaned by sequentially sonicating in acetone (Sigma-Aldrich; ACS reagent, ≥99.5%) and isopropanol (Sigma-Aldrich; ACS reagent, ≥99.5%), blow-dried with nitrogen gas, treated in a UV/O_3_ cleaner (Samco UV-1) at 150 °C for 5 min, and placed at the required downstream position from the furnace center.

### Mn^2+^-Doped (BA)_2_PbBr_4_ (BAPB) Nanoplatelet (NPL) Growth

Upon placing the substrate at 14.5 cm away toward the downstream end from the center, PbBr_2_ (Thermo-Scientific Chemicals; 99.998% (metals basis)) and MnBr_2_ (Thermo-Scientific Chemicals; 99% anhydrous) powders were placed in two separate quartz source boats at the center of a single zone furnace (Fisher Scientific; Lindberg/Blue M Mini-Mite), and BABr (Sigma-Aldrich; ≥98%) powder was placed at 14.5 cm away from the center to the upstream end (see Figure S1A for a detailed diagram). Various molar ratios of MnBr_2_/PbBr_2_ precursors (σ) were used to achieve controlled Mn^2+^ substitution in the crystals, as detailed in the main text. Prior to the reaction, the quartz tube was baked at 950 °C for 1 h with 50 standard cubic centimeters per minute (sccm) of argon (Ar) gas under a vacuum and was allowed to cool down to room. After loading substrates and precursors, pressure was raised to the 250 Torr with a constant 70 sccm Ar flow, and temperature was raised at 40 °C/min ramp rate to 350 °C (temperature at the furnace center *T* ^0 cm^) for NPL growth. The reaction was allowed to run for 1 h and terminated by opening the furnace for rapid cooling, while the pressure and Ar flow were maintained until the temperature dropped below 100 °C. An exemplary graph of growth parameters recorded during a reaction is presented in Figure S2.

### Mn^2+^-Doped HA_2_PbBr_4_ (HAPB) NPL Growth

Following a procedure similar to our BAPB NPL synthesis, we used hexylammonium bromide (HABr) powder (Sigma-Aldrich; ≥98%) positioned at 13.5 cm toward the upstream end of the reaction chamber. We adjusted the substrate position to 15.5 cm toward the downstream end while maintaining all other reaction conditions identical to those employed in our BAPB NPL growth protocol (see Figure S1B for a detailed diagram)

### Mn^2+^-Doped CsPbBr_3_ Growth

PbBr_2_, MnBr_2_, and CsBr (Thermo-Scientific Chemicals; 99% (metals basis)) powders were placed in a quartz source boat at the center of a single-zone tube furnace. After loading the substrate (at 12 cm downstream from the center) and precursors, pressure was maintained at 0.13 Torr with a constant 100 sccm Ar flow, and temperature was raised to 420 °C. The reaction was allowed to run for 30 min. Heating was then stopped, and the sample was allowed to cool to 300 °C with the furnace closed. The furnace was then opened to an Ar flow until the sample temperature dropped below 250 °C (see Figure S1C for a detailed diagram)

### Structural and Elemental Analysis

Powder X-ray diffraction was obtained on a Bruker D8 advance diffractometer using Cu Kα radiation (λ = 1.5418 Å) having a LYNXEYE (1D mode) detector over the 2θ range of 5–50° with a step size of 0.02° for 15 min.

Atomic force microscopy (AFM) was performed using a Dimension Icon with ScanAsyst from Bruker in tipping mode using silicon tips on a nitride lever from Bruker AFM probes (spring constant *k*: 0.4 N/m).

Scanning electron microscopy (SEM) images were acquired using a JEOL 7500F with a cold field emission emitter in secondary electron detection mode operating at 15.0 kV accelerating voltage and emission current of 10 μA. The JEOL 7500F was equipped with an Oxford Energy-Dispersive Spectroscopy (EDS) system that was used for elemental analysis and mapping.

The scanning transmission electron microscopy (STEM) was performed using a Thermo Fisher Talos F200X G2 instrument. An accelerating voltage of 50 kV was used for imaging. The STEM point resolution was 0.25 nm, with a line resolution of 0.12 nm and an information limit <0.12 nm with 0.10 nm attainable. Lacey carbon, 300 mesh, gold, with an approximate grid hole size of 63 μm grid was used for the STEM and high-angle annular dark-field (HAADF) measurements.

### Optical Characterization

Optical images were captured using a Zeiss upright optical microscope (Axio Imager A2m). Dark-field (DF) images were acquired using micro-LED light, and PL images were taken using a UV LED flashlight (λ=365 nm) as an excitation source. The photoluminescence (PL) study was conducted by using a HORIBA Fluorolog-QM with a 75 W xenon arc lamp as the excitation source. For steady-state PL spectra measurements, samples were excited at 370 nm with 4 nm excitation and emission slit widths. For PL excitation spectra, (BA)_2_Mn_*x*_Pb_1–*x*_Br_4_ with *x* = 0 at an emission wavelength of 430 nm and with *x* = 0.045 at 614 nm was measured. Time-resolved PL was measured using HORIBA time-correlated single-photon counting (TCSPC) components with a 373 nm picosecond laser diode (Delta Diode 375L) pulsed at 10 MHz; emission was set to 415 nm for (BA)_2_Mn_*x*_Pb_1–*x*_Br_4_ with *x* = 0 and to 413 nm with *x* = 0.045, with a 2 nm emission slit width. Time-resolved PL spectra of (BA)_2_Mn_0.045_Pb_0.955_Br_4_ were recorded using a Photon Technologies International (PTI) QuantaMaster spectrofluorometer (QM-400), excited at 400 nm and detected at 600 nm, with a lamp frequency of 100 Hz and a total of 8000 shots.

### Power-Dependent PL

Power-dependent PL was measured using a Leica Stellaris 5 confocal microscope with a Leica 63× HC PL APO OIL (1.40NA) objective lens at the Center for Advanced Microscopy at MSU. The sample was excited with a 405 nm continuous wave laser, and emission was collected between 410 and 800 nm using a HyD S2 detector. The pixel dwell time was set to 3.1625 μs, and the scan speed was 400 Hz. A series of spectral images were acquired with 10 nm bandwidth and 5 nm step size. The laser spot size was determined using the theoretical XY optical resolution (*d*_*xy*_ = 0.61λ/NA). Laser power was measured after the objective lens using a photodiode detector (918D-SL, Newport) with an optical power meter (1936-R, Newport).

### Magnetic Characterization

Electron paramagnetic resonance (EPR) measurements were made using a Bruker EMX-plus spectrometer operating at the X-band using an ER-4119HS resonator. Sample temperatures were maintained by using a modified Bruker liquid nitrogen temperature controller. The data shown in Figure 5A were collected using the following parameters: microwave frequency, 9.31 GHz; microwave power, 2 mW; field modulation amplitude, 100 kHz; and sample temperature, 130 K.

All of the magnetic moment measurements were taken by using the Quantum Design MPMS3 superconducting quantum interference device (SQUID) magnetometer. We had NPLs of (BA)_2_Mn_0.045_Pb_0.955_Br_4_ with 70 nm thickness on silica mounted on the MPMS 3 Quartz Paddle sample holder with the help of a Kapton tape and then inserted it into the magnetometer. All of the magnetic fields were applied parallel to the thin film. For the *M*–*H* loop, we applied the magnetic field from −7 to 7 T by keeping the temperature constant. In the *M*–*T* curve, the sample was cooled to 2.5K under zero-field-cooled conditions first, and then the moment was measured from 2.5 to 300 K under 7 T external applied field.

### Theoretical Calculations

First-principles density functional theory calculations were performed using the projector-augmented-wave method.^76^ and the local density approximation, as implemented in the Vienna *Ab initio* Simulation Program code.^77^ The 6s and 6p electrons of Pb, 4s and 4p electrons of Br, 3d and 4s electrons of Mn, 2s and 2p electrons of C, 2s and 2p electrons of N, and 1s electron of H were used as valence electrons. A plane-wave basis cutoff energy was set to 800 eV. The *k*-point meshes of 2 × 2 × 1 and 8 × 8 × 2 were employed for the structural relaxation and electronic structure calculations, respectively. An electronic self-consistency convergence criterion of 1 × 10^–6^ eV was used. The crystal orbital Hamilton population analysis was performed using the Local-Orbital Basis Suite Toward Electronic-Structure Reconstruction package.^78–80^ The crystal structure was visualized using the VESTA code.^81^

## Supplementary Material





## Notes

Additional experimental and calculated results (PDF)
Raw data tables for published results (XLSX)
