# Supplementary material for: Deterministic Structural Distortion in Mn2+-Doped Layered Hybrid Lead Bromide Perovskite Single Crystals
Source: ACS Nano. 2025 Jul 17;19(29):26920–31. doi: 10.1021/acsnano.5c08324 (PMC12312163; doi:10.1021/acsnano.5c08324)
Supplement: Supplementary file 1 [file nn5c08324_si_001.pdf]

Supporting Information for:

Deterministic Structural Distortion in Mn^{2+}-doped Layered Hybrid Lead Bromide Perovskite Single Crystals

Pushpender Yadav,[1†] Kyeongdeuk Moon,[1†] Muhammad Shoaib,[1] Puja Thapa,[2] Rui Sun,[2] Seungmin Yang,[3] Jung-Moo Heo,[4] Sijun Seong,[5] John McCracken,[1] Xiwen Gong,[4,5] Jinsang Kim,[4] Joonho Bang,[3,6] Dali Sun,[2] Seokhyoung Kim[1]

[1]*Department of Chemistry, Michigan State University, East Lansing, MI 48824, USA*
[2]*Department of Physics, North Carolina State University, Raleigh, NC 27695, USA*
[3]*Department of Materials Engineering and Convergence Technology, Gyeongsang National University, Jinju 52828, South Korea.*
[4]*Department of Material Science and Engineering, University of Michigan, Ann Arbor, MI 48109, USA*
[5]*Department of Chemical Engineering, Department of Electrical Engineering and Computer Science, Macromolecular Science and Engineering Program, Applied Physics Program, University of Michigan, Ann Arbor, MI 48109, USA*
[6]*School of Materials Science and Engineering, Gyeongsang National University, Jinju 52828, South Korea.*

[†]*Equal contribution*

Supporting information includes:

Figure S1: Schematic diagram of CVD depicting precursor and substrate loading

Figure S2: Recorded experimental growth parameters

Figure S3: Additional optical images of Mn^{2+}-doped BAPB

Figure S4: AFM scans of Mn^{2+}-doped BAPB

Figure S5: Single NPL EDS analysis of Mn^{2+}-doped BAPB

Figure S6: x vs γ analysis of Mn^{2+}-doped BAPB NPLs for $\sigma = 0.03$

Figure S7: x vs γ analysis of Mn^{2+}-doped BAPB NPLs for $\sigma = 0.15$

Figure S8: x vs γ analysis of Mn^{2+}-doped BAPB NPLs for $\sigma = 0.50$

Figure S9: Cell parameters 'a' and 'b' of Mn^{2+}-doped BAPB for $x = 0.045$

Figure S10: Optical images of Mn^{2+}-doped $CsPbBr_3$

Figure S11: Optical images of Mn^{2+}-doped HAPB

Figure S12: XRD of Mn^{2+}-doped HAPB

Figure S13: Mn^{2+}-doped BAPB NPLs on different substrates for σ = 0.50

Figure S14: Projected density of states (DOS) for Mn^{2+}-doped BAPB

Table S1: Lattice parameters of BAPB with/without Mn^{2+}-substitution.

Table S2: Fitting parameters of undoped and doped BAPB PL decay plot.

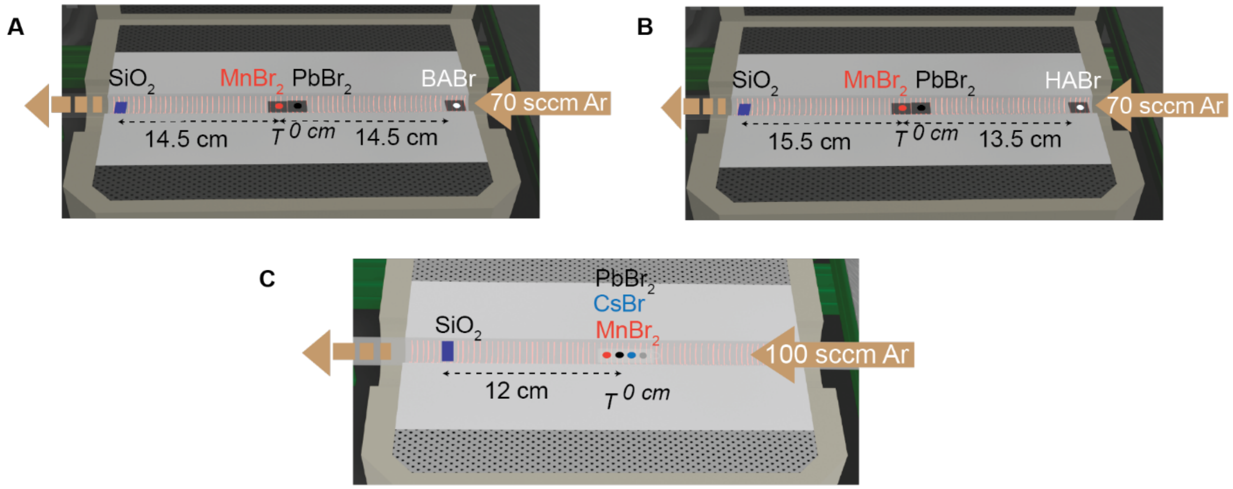

Figure S1 | Schematic diagram of CVD depicting precursor and substrate loading. (A) Mn^{2+}-doped BA_2PbBr_4 NPLs, (B) Mn^{2+}-doped HA_2PbBr_4 NPLs, (C) Mn^{2+}-doped $CsPbBr_3$ growth.

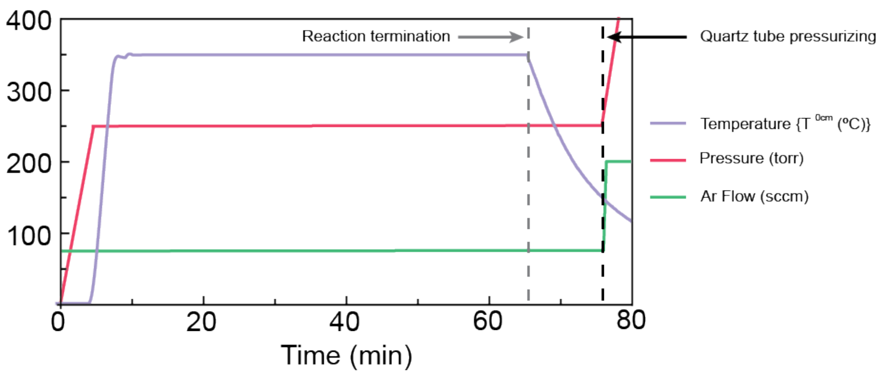

Figure S2 | Recorded experimental growth parameters. Plots of T^{0cm}, reaction pressure, and Ar flow rate recorded real-time during a CVD run with reaction termination time by opening the furnace (gray dotted line), opening of quartz tube time to take substrate out of reaction chamber (black dotted line).

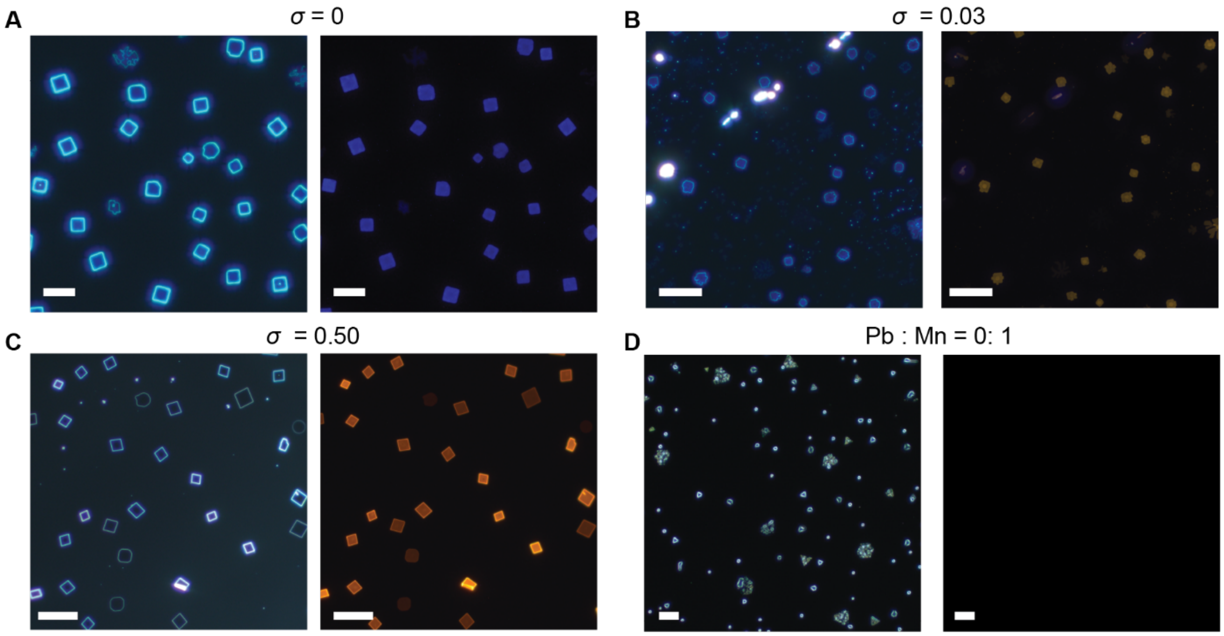

Figure S3 | Additional optical images of Mn^{2+}-doped BAPB. DF (left) and PL (right) images for $\sigma = 0$ (A), 0.03 (B), 0.50 (C), and fully Mn^{2+} substituted NPLs (D); scale bars, 10 μm.

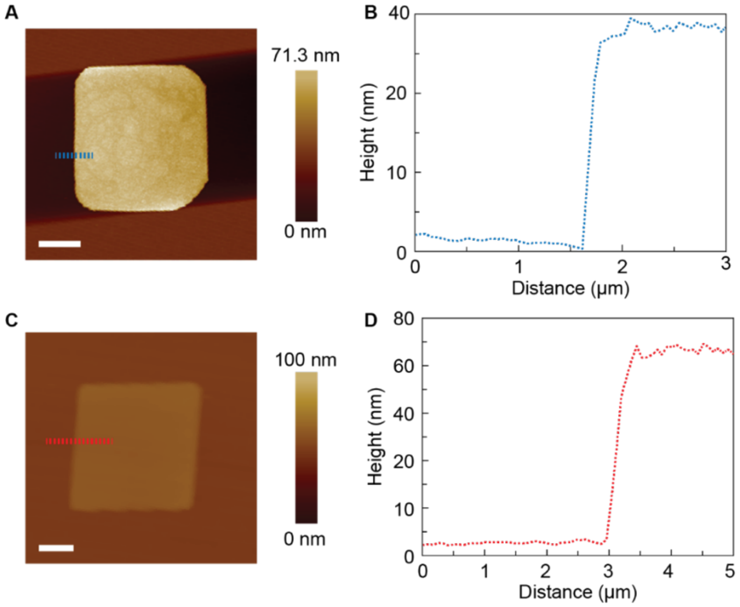

Figure S4 | AFM scans of Mn^{2+}-doped BAPB. For σ = 0 AFM topology map (A), step heigh scan (B), σ = 0.05 AFM topology map (C) step heigh scan (D); scale bars, 2 µm.

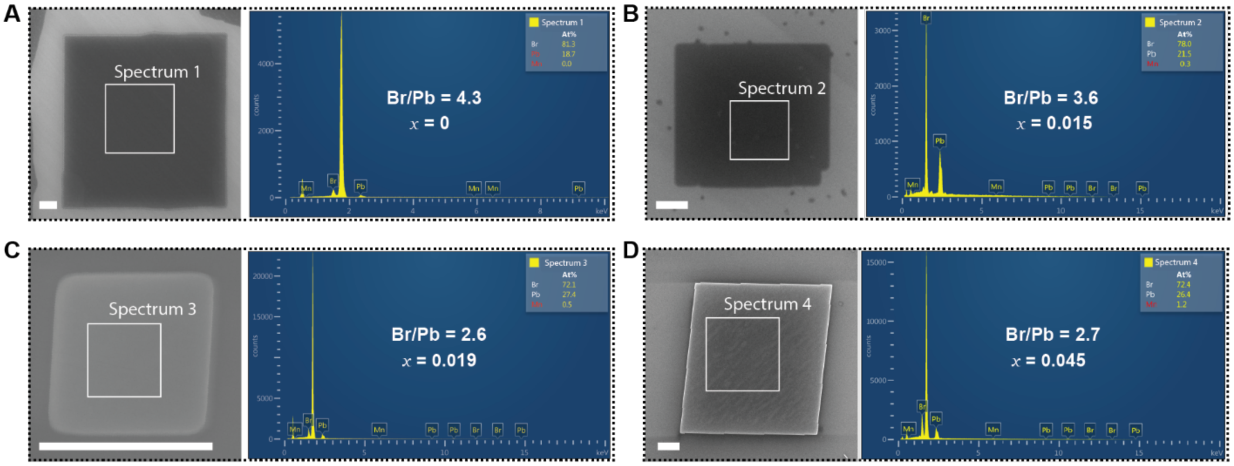

Figure S5 | Single NPL EDS analysis of Mn^{2+}-doped BAPB. SEM images (left) and EDS scans (right) for σ = 0 (A), 0.03 (B), 0.15 (C), and 0.50 (D); scale bars 1 µm.

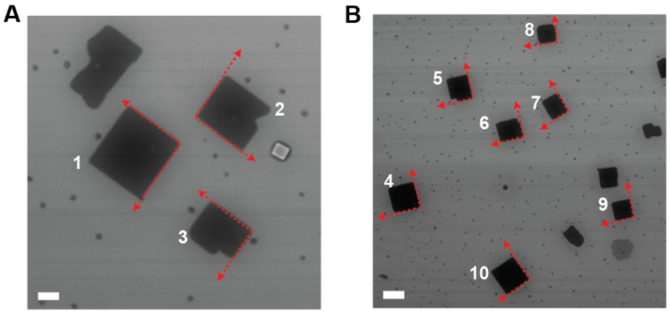

C

NPL	x (×10^{-2})	γ(°)
1	1.23	90.14
2	0.79	90.35
3	0.74	90.55
4	1.12	90.41
5	1.39	90.22
6	0.71	90.21
7	1.02	90.33
8	1.03	90.25
9	1.35	90.66
10	1.05	90.29

Figure S6 | x vs γ analysis of Mn^{2+}-doped BAPB NPLs for σ = 0.03. (A-B) SEM images with red dotted lines representing **γ**, (B) Table containing x and **γ** values for 10 different NPLs; scale bars 1 µm.

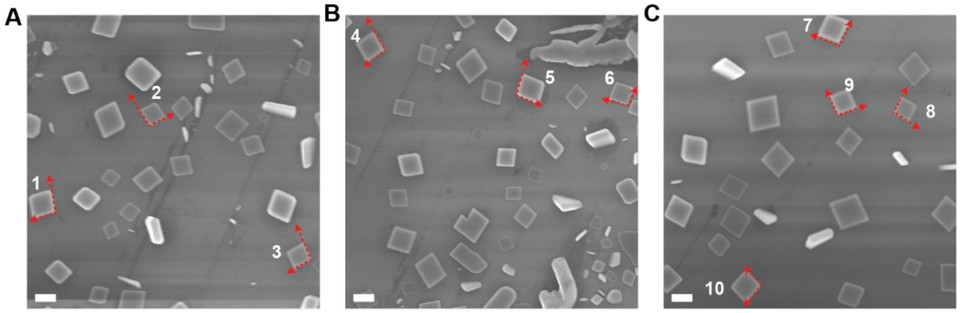

D

NPL	x (×10^{-2})	γ(°)
**1**	2.06	93.50
**2**	1.35	92.73
**3**	1.40	92.86
**4**	1.68	93.15
**5**	2.05	93.44
**6**	1.69	93.36
**7**	2.47	93.78
**8**	1.78	93.56
**9**	1.71	93.44
**10**	1.81	93.73

Figure S7 | *x* vs γ analysis of Mn^{2+}-doped BAPB NPLs for *σ* = 0.15. (A-C) SEM images with red dotted lines representing **γ**, (D) Table containing *x* and **γ** values for 10 different NPLs; scale bars 1 μm.

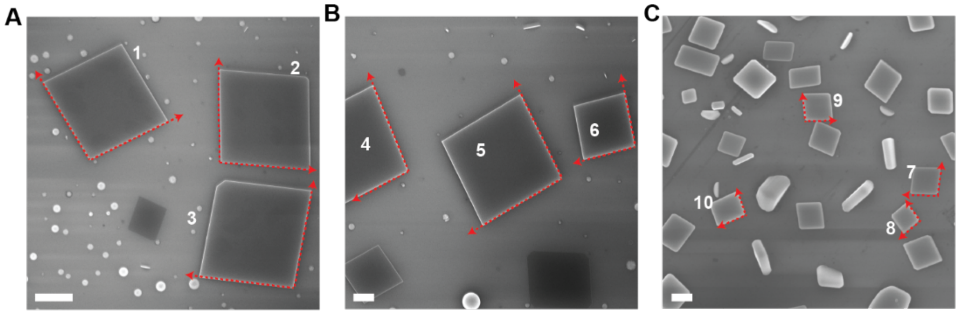

D

NPL	x (×10^{-2})	γ(°)
**1**	4.50	95.61
**2**	4.54	95.65
**3**	4.95	95.72
**4**	4.15	94.17
**5**	4.51	94.83
**6**	4.54	94.97
**7**	4.84	94.91
**8**	4.08	94.57
**9**	4.14	94.86
**10**	4.51	95.52

Figure S8 | *x* vs γ analysis of Mn^{2+}-doped BAPB NPLs for *σ* = 0.50. (A-C) SEM images with red dotted lines representing **γ**, (D) Table containing *x* and **γ** values for 10 different NPLs; scale bars 1 μm.

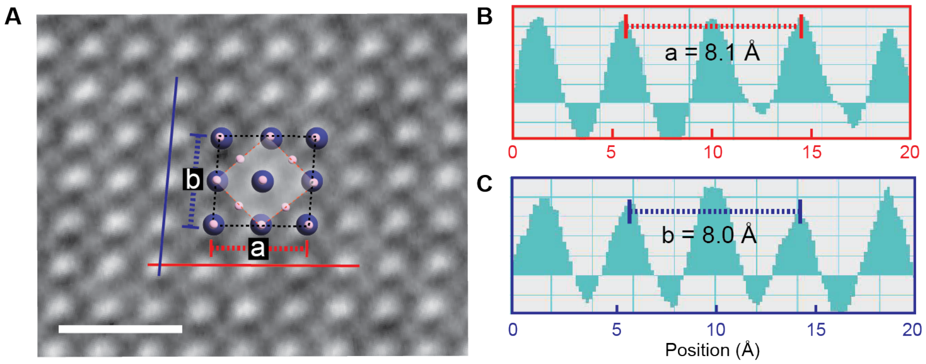

Figure S9 | Cell parameters 'a' and 'b' of Mn^{2+}-doped BAPB for x = 0.045. (A) STEM-HAADF atom-resolved image of x = 0.045 NPL with projected unit cell on top and line scans for bond distance; scale bar: 1 nm. (B, C) projected bond distance analysis of 'a' in red dotted line (B), 'b' in blue dotted line (C).

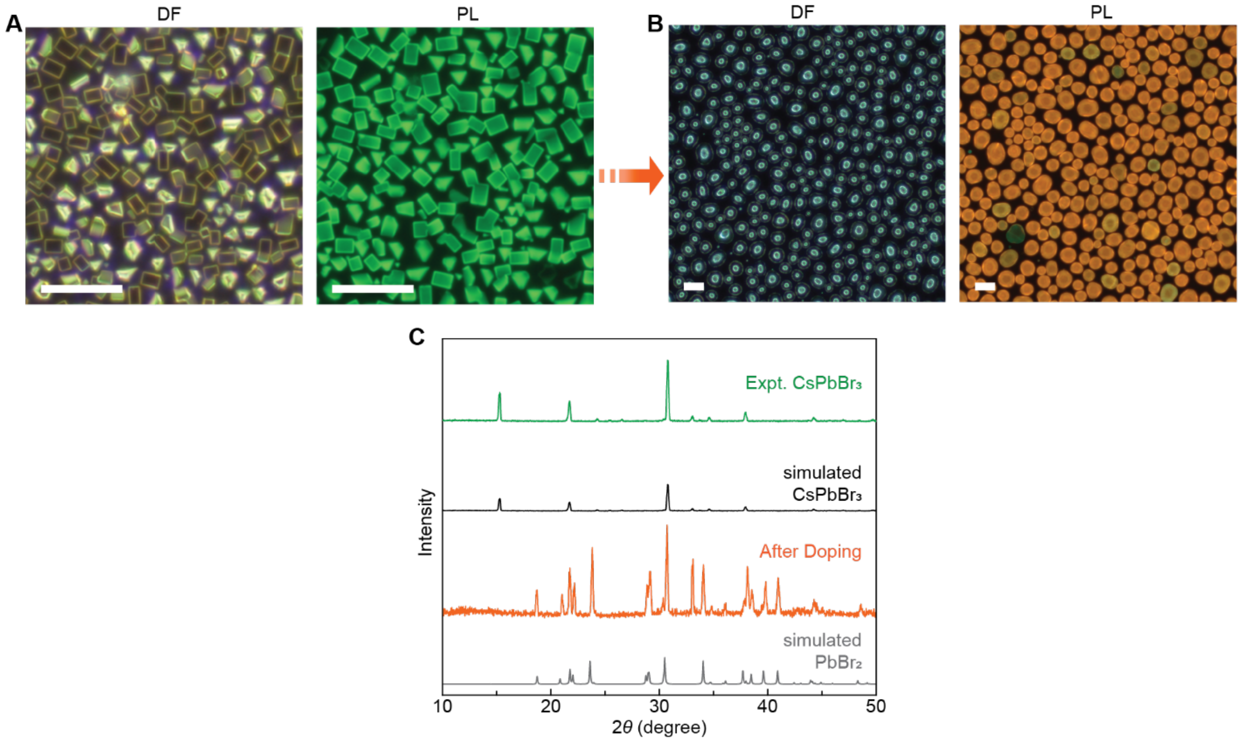

Figure S10 | Optical images of Mn^{2+}-doped $CsPbBr_3$. DF (left) and PL (right) images for undoped $CsPbBr_3$ (A), attempted Mn^{2+} doping for $CsPbBr_3$ (B), XRD patterns (C); scale bars, 50 μm.

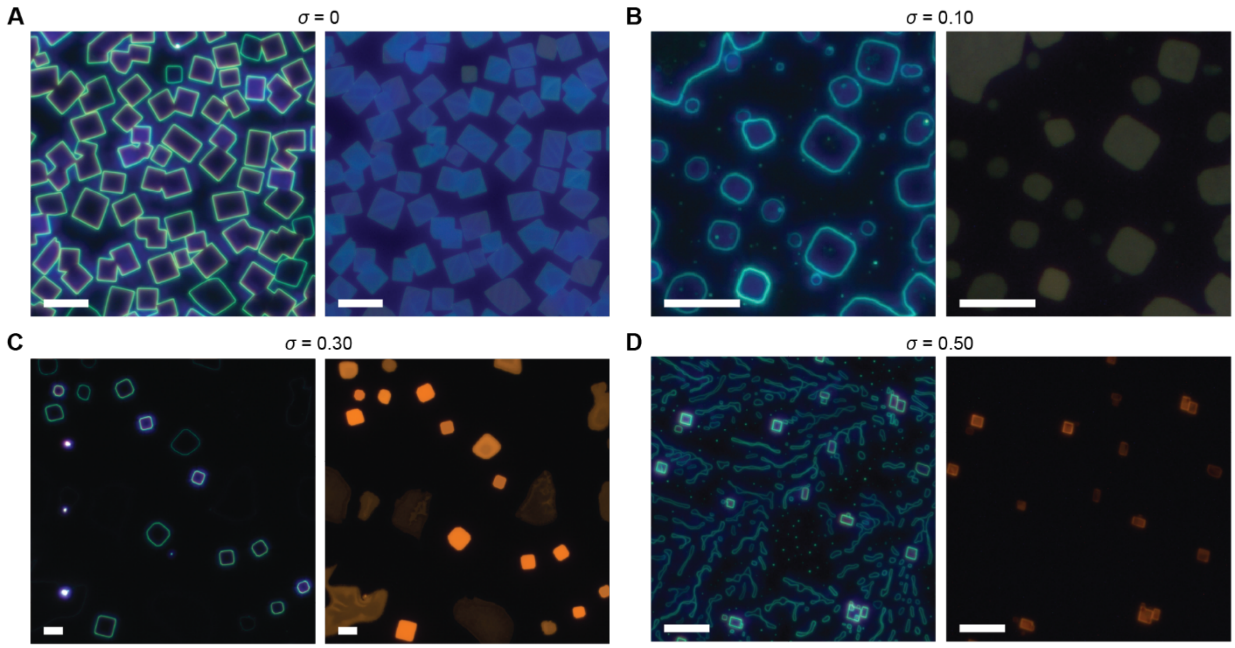

Figure S11 | Optical images of Mn^{2+}-doped HAPB. DF (left) and PL (right) images for $\sigma = 0$ (A), 0.10 (B), 0.30 (C), and 0.50 (D); scale bars, 10 μm.

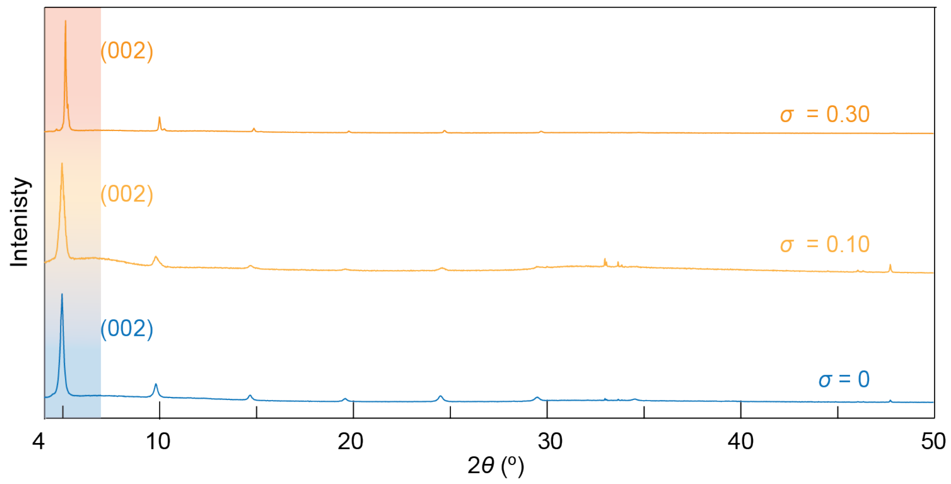

Figure S12 | XRD of Mn^{2+}-doped HAPB.

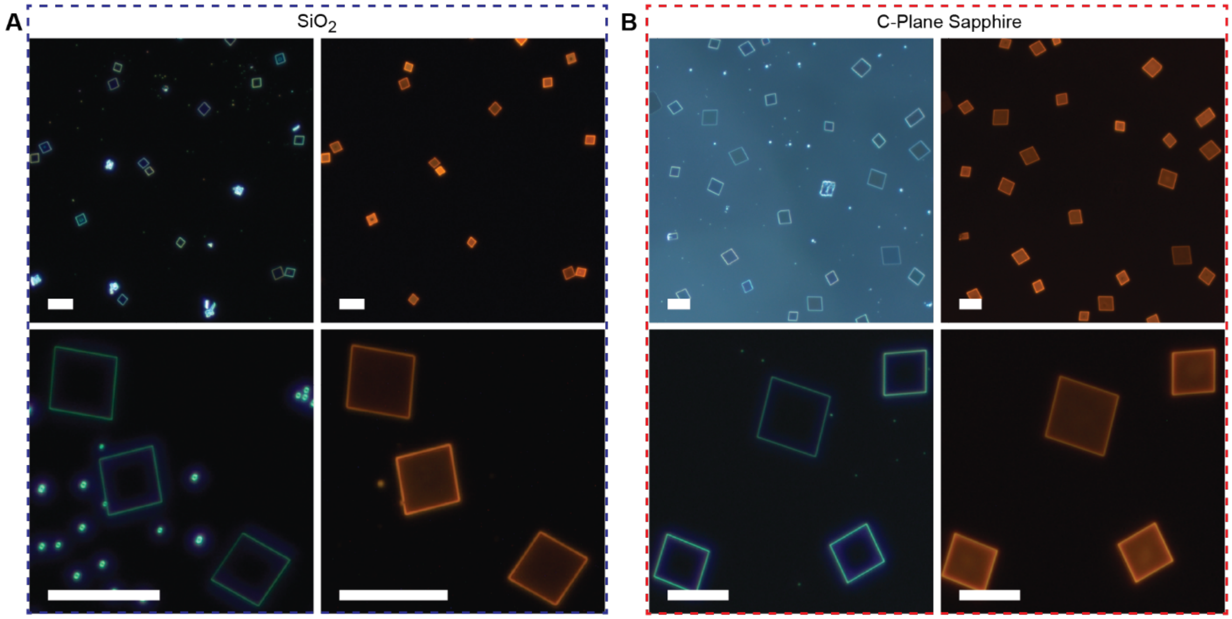

Figure S13 | Mn^{2+}-doped BAPB NPLs on different substrates for σ = 0.50. DF (left) and PL (right) images at low (top) and high (bottom) magnification on SiO_2 (A), C-plane sapphire (B) substrate; scale bars, 10 μm.

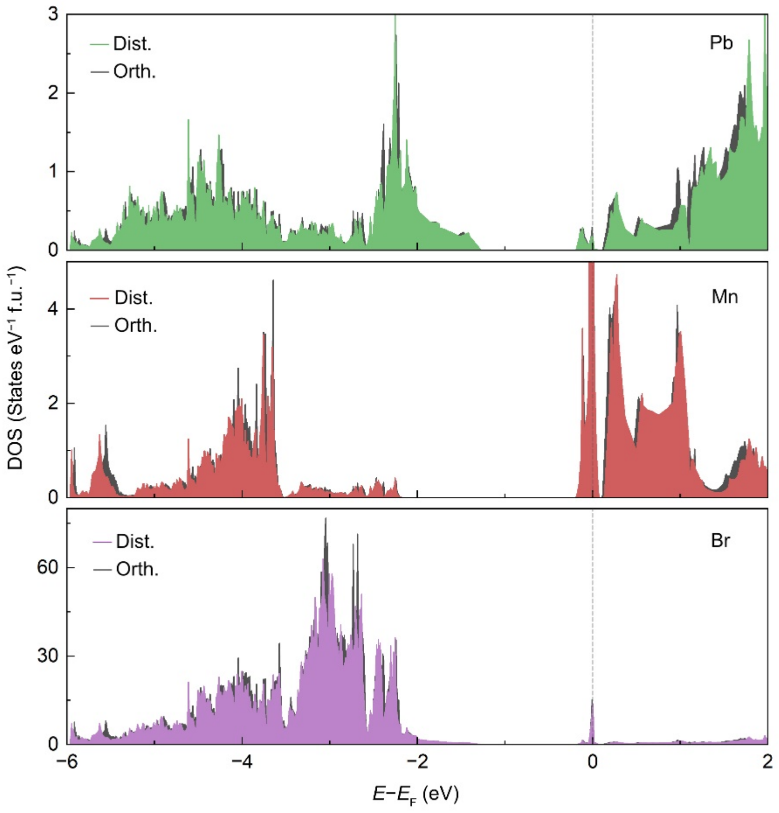

Figure S14 | Projected density of states (DOS) for Mn^{2+}-doped BAPB. The projected DOS of partially Mn^{2+}-substituted BAPB for the distorted (Dist.) and non-distorted orthorhombic (Orth.) structures.

Table S1 | DFT-calculated lattice parameters of BAPB with/without Mn^{2+}-substitution.

	Structure	a(Å)	b(Å)	c(Å)	α(°)	β(°)	γ(°)	V(Å^3)
Undoped	Orthorhombic	7.966	7.933	26.256	90.0	90.0	90.0	1659.1
Doped	Orthorhombic	7.903	7.820	26.048	90.0	90.0	90.0	1620.1
	Distorted	7.840	7.765	26.218	91.1	89.5	89.9	1595.8

Table S2 | Fitting parameters of undoped and doped BAPB PL decay plot.

	τ_1	a_1 (%)	τ_2	a_2 (%)
Undoped @ 400 nm	0.9 ns	92	1.6 ns	8
Doped @ 400 nm	0.3 ns	95	3.2 ns	5
Doped @ 600 nm	7.2 μs	89	378 μs	11
